# In-Depth Immune-Oncology Studies of the Tumor Microenvironment in a Humanized Melanoma Mouse Model

**DOI:** 10.3390/ijms22031011

**Published:** 2021-01-20

**Authors:** Jonathan Schupp, Arne Christians, Niklas Zimmer, Lukas Gleue, Helmut Jonuleit, Mark Helm, Andrea Tuettenberg

**Affiliations:** 1 Department of Dermatology, University Medical Center, Johannes Gutenberg University Mainz, 55131 Mainz, Germany; jonathan.schupp@kgu.de (J.S.); Niklas.Zimmer@unimedizin-mainz.de (N.Z.); jonuleit@uni-mainz.de (H.J.); 2 Zellkraftwerk GmbH, 04103 Leipzig, Germany; Arne.Christians@bruker.com; 3 Institute of Pharmacy and Biochemistry, Johannes Gutenberg University Mainz, 55128 Mainz, Germany; lugleue@uni-mainz.de (L.G.); mhelm@uni-mainz.de (M.H.)

**Keywords:** Chipcytometry, multiplex immunohistochemistry, melanoma, humanized mice, tumor microenvironment, flow cytometry, immunohistochemistry

## Abstract

The presence and interaction of immune cells in the tumor microenvironment is of significant importance and has a great impact on disease progression and response to therapy. Hence, their identification is of high interest for prognosis and treatment decisions. Besides detailed phenotypic analyses of immune, as well as tumor cells, spatial analyses is an important parameter in the complex interplay of neoplastic and immune cells—especially when moving into focus efforts to develop and validate new therapeutic strategies. Ex vivo analysis of tumor samples by immunohistochemistry staining methods conserves spatial information is restricted to single markers, while flow cytometry (disrupting tissue into single cell suspensions) provides access to markers in larger numbers. Nevertheless, this comes at the cost of scarifying morphological information regarding tissue localization and cell–cell contacts. Further detrimental effects incurred by, for example, tissue digestion include staining artifacts. Consequently, ongoing efforts are directed towards methods that preserve, completely or in part, spatial information, while increasing the number of markers that can potentially be interrogated to the level of conventional flow cytometric methods. Progression in multiplex immunohistochemistry in the last ten years overcame the limitation to 1–2 markers in classical staining methods using DAB with counter stains or even pure chemical staining methods. In this study, we compared the multiplex method Chipcytometry to flow cytometry and classical IHC-P using DAB and hematoxylin. Chipcytometry uses frozen or paraffin-embedded tissue sections stained with readily available commercial fluorophore-labeled antibodies in repetitive cycles of staining and bleaching. The iterative staining approach enables sequential analysis of a virtually unlimited number of markers on the same sample, thereby identifying immune cell subpopulations in the tumor microenvironment in the present study in a humanized mouse melanoma model.

## 1. Introduction

The tumor microenvironment (TME) is a complex network of interactions between immune cell populations, cancer cells, and vascular and stromal components, which play a critical role in cancer cell growth, disease, prognosis, and therapeutic approaches. Beside detailed phenotypic analysis of cell types in the tumor microenvironment, including immune, as well as stroma and tumor cells, spatial analysis is also important to unravel the complex interplay of neoplastic and immune cells with and without therapy and to develop and validate new therapeutic strategies [1,2,3,4].

Whereas in vitro systems allow functional analysis on a single cell level, the complex interplay of immune reactions in the TME and especially the investigation of new therapeutic approaches warrant (i) an efficient in vivo system and (ii) suitable monitoring of the TME. In the present study, we used inoculation of immunodeficient mice with a melanoma cell line to establish in situ immunomonitoring with the future goal to validate the effects of different therapeutic approaches in vivo. Although the use of tumor models in immunocompetent mice can indicate biologic activity of different therapies, many approaches, such as therapies using monoclonal antibodies (mAb), are species- and epitope-specific and require the expression of human receptors. Thus, the utilization of humanized mouse models seems to be reasonable. Several groups used immunodeficient mice transplanted with human tumors and subsequent transfer of human immune cells and therapeutic mAb [5,6,7]. Although anti-tumor responses can be assessed in these systems, xenoreactivity, and onset of graft-versus-host disease (GvHD) conceal which cellular component or immunological event mediates observed effects. Advanced humanized mouse systems, including transgenic expression of relevant human molecules in immunodeficient mice, are a possible solution to improve the robustness of these models [8]. Expression of human major histocompatibility complex (MHC) class I and/or II molecules on murine cells possibly reduce xenoreactivity and delay onset of GvHD mediated by human leukocyte antigen (HLA)-matched CD8^+^ and CD4^+^ T cells.

Thus, in our study, we used immunodeficient NOD/Scid mice transgenic for HLA-A2 molecules as in vivo model system with the focus to perform in-depth immunomonitoring to analyze the tumor micromilieu using different approaches, such as flow cytometry, multiplex analysis, and immunohistochemistry.

Using flow cytometry, multiple parameters can be detected on the cell surface and in other cellular compartments [9]. However, concrete localization of cells within the tumor tissue cannot be analyzed using this technique as for analysis, tissues need to be disrupted into single cell suspensions. Additionally, flow cytometry lacks certain flexibility. Samples have to be run in real-time (within 1–3 days). This does not allow for storage and repeated analysis of samples—which is especially important when using valuable clinical study samples. The set of markers for analysis needs to be predefined and has to be established before the sample is processed. Thus, flow cytometry cannot be easily adjusted if new clinical questions or hypotheses arise during the period of analysis or the long term follow up of patients. In addition, as tumor tissue has to be digested before analysis, the cellular content may be affected in general, making it difficult to obtain sufficient cell numbers for detailed analysis. Furthermore, cell viability can be affected through sample preparation methods leading to a rapid decline in cell numbers and viability after sample collection.

Conventional IHC is commonly used for diagnostic issues conserving spatial information, but suffers from certain limitations, such as only permitting the labeling of single markers on one tissue section. So far, the number of cluster of differentiation (CD) markers that can be assessed on one tissue section by routine immunohistochemistry (IHC) is limited, making multiplex analysis, particularly of rare cells with a distinct phenotype, such as regulatory T cells or macrophages difficult. Often it is not possible to stain human immune cell subpopulations in tissue samples with the full marker profile needed for detailed phenotyping.

In contrast, multiplex-immunofluorescence is a technique that strains more than 100 biomarkers consecutively on the same cell [10,11].

Slide-based cytometry was first introduced as an alternative to flow cytometry. This method is based on automated epifluorescence microscopy of cells that are immobilized on a solid surface. Since then, this concept has been enhanced with iterative restaining and photobleaching of fluorochromes as newly introduced components. The herein used platform (Chipcytometry) is a modified approach to slide-based cytometry developed at the Hannover Medical School [12]. Based on microfluidic chips containing cell-adhesive surfaces that allow long-term storage and repeated staining and washing steps by simple fluid exchange, Chipcytometry allows for iterative multiplex immunofluorescence staining and analysis of a virtually unlimited number of biomarkers on a single tissue section, including cell–cell interactions and cellular functions. AI-based cell recognition of stained cells allows for the generation of quantitative, “flow-like” single cell data, while also considering the spatial information of tissue structure, like in classical immunohistochemistry methods. As the tissue is preserved during and after the staining procedure, the sample can be stored and re-analyzed for up to 20 months with additional markers at any time, even if new or additional questions are to be addressed.

So far, Chipcytometry has been applied several times, especially to study circulating tumor cells, cerebrospinal fluid cells, and mesenchymal stromal cells [13,14,15]. Nevertheless, recently, this technique has been proved to be also suitable to analyze tissue samples originating from the colon, lung, and brain [16,17,18]. Of note, skin cancer, especially melanoma, has not been studied so far. Whereas the treatment success with checkpoint inhibitors, especially in melanoma, has been ground-breaking both for progression-free and overall survival, only part of the patients show a good response, whereas others do not benefit from this treatment. Many efforts are made to investigate biomarkers to identify and characterize parameters in peripheral blood or tumor microenvironment from melanoma patients to solve this question [19]. In the present study, we investigated immunophenotyping of melanoma, and report on its comparison to flow cytometry and IHC in a relevant preclinical humanized mouse model of melanoma.

Being aware that the three different methods described here are different in the preparation of tissues and cells, the present study wants to point out the advantages and disadvantages of every single method and its possible application in preclinical and clinical studies to study the TME. Whereas the standard immune histochemistry allows the detection and co-localization of cells and is a cost- and time effective and applicable method for standard diagnostic although displaying a high inter-observer variability, mostly only a few markers are stained at a time [11] In contrast, flow cytometry allows a broader panel of phenotypic markers and detailed analysis of cellular subpopulations. Nevertheless, after tissue sample preparation, spatial information and cellular co-localization are lost. Herein, a modified multiplexed approach to slide-based cytometry might be a useful and complementary tool for analysis of tumor tissue, due to the possibility of iterative staining, imaging, and bleaching cycles, especially in the context of translational research.

## 2. Results

### 2.1. Humanized Mouse Melanoma Model

**Figure 1:**
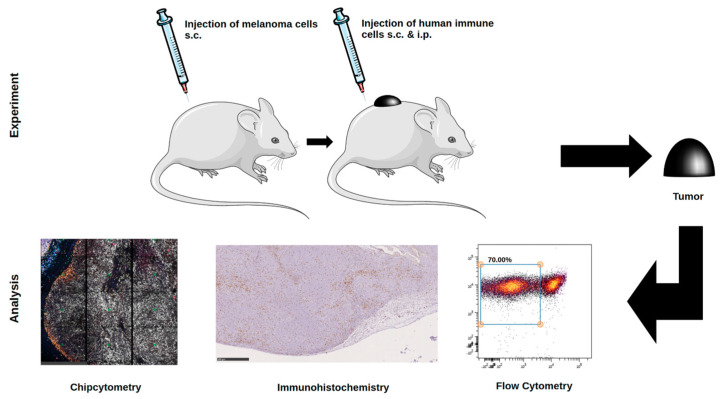
Schematic of the melanoma model in the humanized mice. After subcutaneous injection of human Ma-Mel-19 melanoma cells in NOD Scid tgHLA-A2.1 mice, human immune cells were injected at the tumor site s.c. and i.p. After three more weeks, ex vivo analysis of tumor was performed using Chipcytometry, immunohistochemistry, and flow cytometry.

In the present study, we used the inoculation of immunodeficient mice with a human melanoma cell line grown in the presence of a humanized immune system, as shown before to perform comparative analyses of the tumor microenvironment by multiplex staining, immunohistochemistry, and flow cytometry (Figure 1) [11]. This mouse model is particularly relevant, because tumor progression critically depends on the state of human immune cells. The latter were PBMC preparations from HLA-A2^+^ healthy human donors. Tissues of interest for analysis were the melanomas induced. Characterization of molecular markers in these tissues is of high interest for the characterization of various immunotherapeutic treatments [20,21].

**Figure 2:**
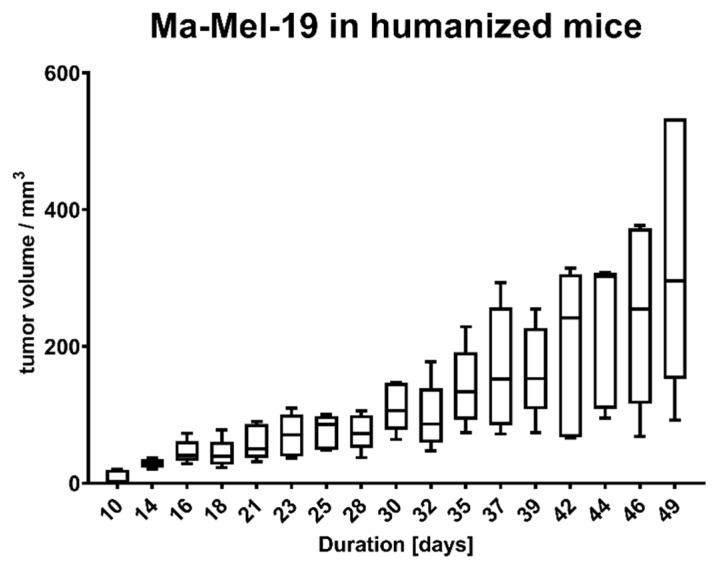
Tumor growth of Ma-Mel-19 cells in humanized mice. Mice were humanized on day 23. Data are shown as box plots with whiskers at min/max. six animals per group.

Human Ma-Mel-19 melanoma cells injected s.c. into the back of immunodeficient NOD Scid tgHLA-A2.1 mice substituted with human immune cells led to the formation of a xenograft tumor over the course of more than 40 days (Figure 2).

### 2.2. Immunomonitoring Using Different Approaches

**Figure 3:**
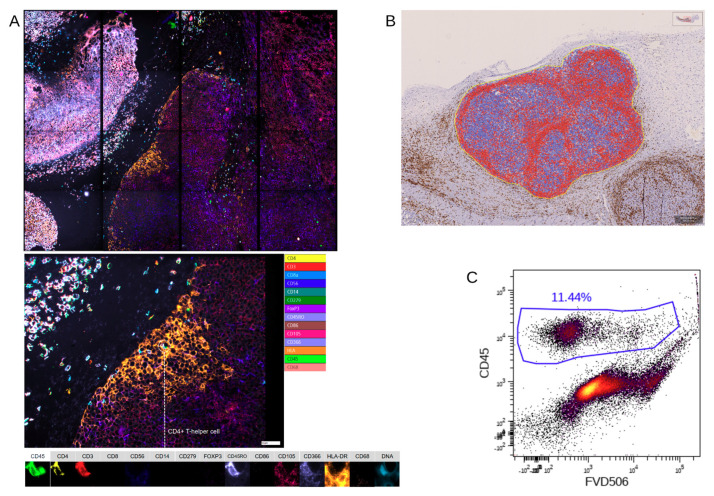
Multimarker analysis of the whole tumor, as well as a single cell in Chipcytometry (**A**), data analysis of CD45^+^ (positive cells in red, negative cells in blue) cells in immunohistochemistry (**B**), and gating for all CD45+ against a live/dead stain, human immune cells in flow cytometry (**C**). One typical experiment is shown.

**Figure 4:**
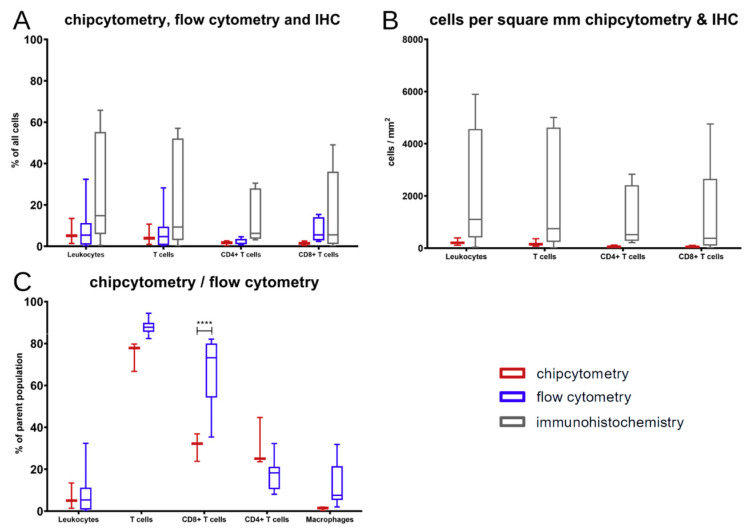
Data comparison of immune cell composition in the tumor microenvironment obtained by Chipcytometry (red), flow cytometry (blue), and immunohistochemistry (grey). Since gating is possible in flow cytometry and Chipcytometry percentages of all cells, respectively, immune cells can be shown (**A**,**C**), whereas spatial information is only preserved in Chipcytometry and IHC (**B**). Data are shown as box plots with whiskers at min/max. **** *p* < 0.0001; Statistical significance was determined using the Šídák’s multiple comparison test.

**Figure 5:**
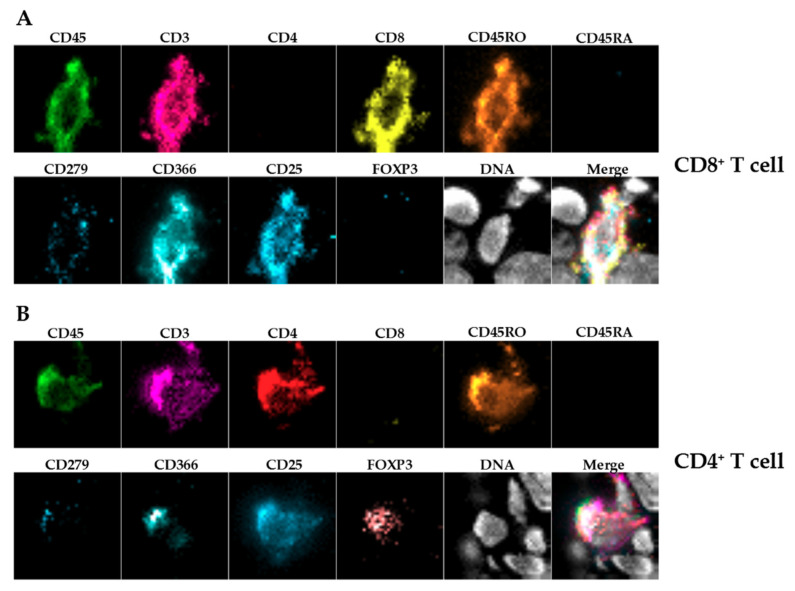
Chipcytometry analysis showing exemplarily the identification of tumor-infiltrating CD8^+^ (**A**) and CD4^+^ (**B**) T cells within their spatial context. The lower right picture shows the merged channels.

In a next step, we compared classical immunohistochemistry to two multiplex methods (Figure 3, Figure A2 and Figure A3). Analysis of the tumor tissue by Chipcytometry, flow cytometry, and IHC-P revealed no significant differences in the broad classification of human immune cells (huCD45^+^) and human T cells (CD45^+^CD3^+^), albeit a high variation in cell percentages was detected by IHC-P (Figure 4A). It is important to mention, that only Chipcytometry and IHC-P can focus exclusively on the tumor mass (Figure 3A,B), whereas in flow cytometry, digestion of the whole tumor sample might lead to detection of PBMC also from the injection site or non-tumor tissue. However, flow cytometry gates on live cells exclusively to avoid false positive marker expression of dying cells or cell remnants (Figure 3C). Further and additional sub-classification into CD3^+^CD4^+^ or CD3^+^CD8^+^ double positive T cells and macrophages (CD45^+^CD68^+^) was only possible by chip and flow cytometry (Figure 4C and Figure 5). There was a significant discrepancy in T cell subset distribution detected when comparing of both methods. Whereas, overall human leukocytes (huCD45^+^), T cells (CD45^+^CD3^+^) and macrophages (CD45^+^CD68^+^) were comparable, in Chipcytometry 30.9% (SD 6.7%) of all T cells were CD8^+^ and 31.1% (SD 11.7%) were CD4^+^. On the contrary, 67.4% (SD 15.2%) of T cells detected by flow cytometry were CD8^+^ and only 17.5% (SD 7.2%) were CD4+. Of note, only three mice could be analyzed via Chipcytometry, due to limitation of material, whereas the sample size in the flow cytometry and IHC-P was ten and twelve, respectively, due to tumor slices with more than one distinct tumor.

Taking spatial information of Chipcytometry and immunohistochemistry into account, the cell number per square millimeter (Figure 4B) is comparable to cells counted as the percentage of all cells (Figure 4A).

Nonetheless, further analysis of immune cell distribution in the tumor mass and its environment, as well as detection of cell–cell contacts, is feasible using these approaches. As an example, further subpopulations of T cells, such as PD-1^+^/TIM-3^+^ or activated CD45RO^+^ T cells, could be classified by Chipcytometry (see Table 1 and Figure 5). Especially the presence and quantification of PD-1^+^ and TIM-3^+^ T cells may be of future importance, with PD-1 and TIM-3 being important targets molecule in current immunotherapeutic approaches. Thus, changes of the cellular composition in the tumor microenvironment with a focus on CD8^+^PD-1^+^ T cells following, for example, anti-PD-1 therapy can be quantified when using multiplex methods such as Chipcytometry in future experimental setups.

In conclusion, we learned from these data that for further and deep investigations of the TME multiplex analysis would be favorable to investigate in more detail, especially the influence of different systemic treatment approaches with immunomodulatory functions on the composition, but also on the spatial distribution of immune cells in the TME.

### 2.3. Immunomonitoring of the Melanoma Tumor Micromilieu after Tasquinimod Treatment

**Figure 6:**
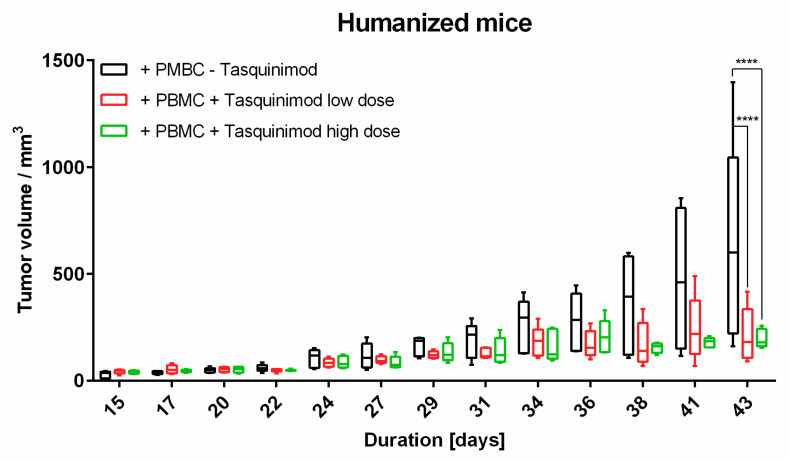
Tumor growth of Ma-Mel-19 cells in humanized mice under treatment with Tasquinimod (high: 5 mg/kg body weight and low: 1 mg/kg body weight). Mice were humanized on day 23. Tasquinimod was applied on day 24, 31, and 37. Data are shown as box plots with whiskers at min/max. **** *p* < 0.0001; Statistical significance was determined using the Tukey’s multiple comparison test. Five animals per group.

Previous reports showed that the immunomodulator Tasquinimod is a small molecule with pleiotropic effects on the tumor microenvironment. Besides its influence on neo-angiogenesis, it can modulate tumor-associated macrophages by switching their rather tolerogenic M2-phenotype into an inflammatory M1-phenotype [22,23]. To analyze the effects of Tasquinimod on immune cells and tumors in vivo using our comparative approach for immunomonitoring, tumor-bearing mice were treated with Tasquinimod in different doses (5 mg/kg body weight (high dose) and 1 mg/kg body weight (low dose)). Weekly treatment significantly hampered tumor growth in both doses applied when compared to untreated mice after three weeks of therapy (Figure 6).

**Figure A1:**
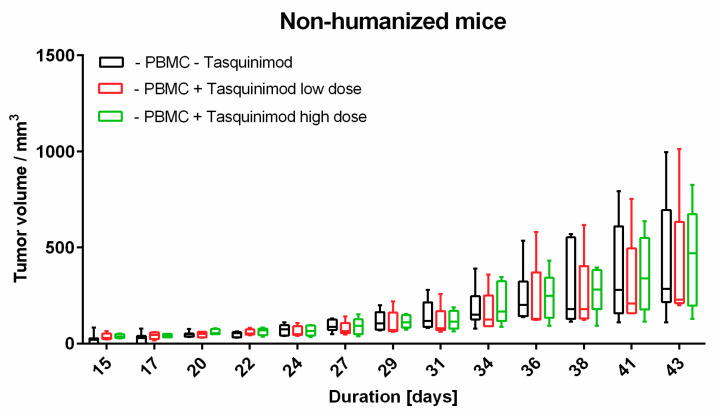
Tumor growth of Ma-Mel-19 cells in immunodeficient non-humanized mice under treatment with Tasquinimod. Tasquinimod was applied on day 24, 31, and 37. Data are shown as box plots with whiskers at min/max. seven animals in control group, five animals in both treated groups.

**Figure A2:**
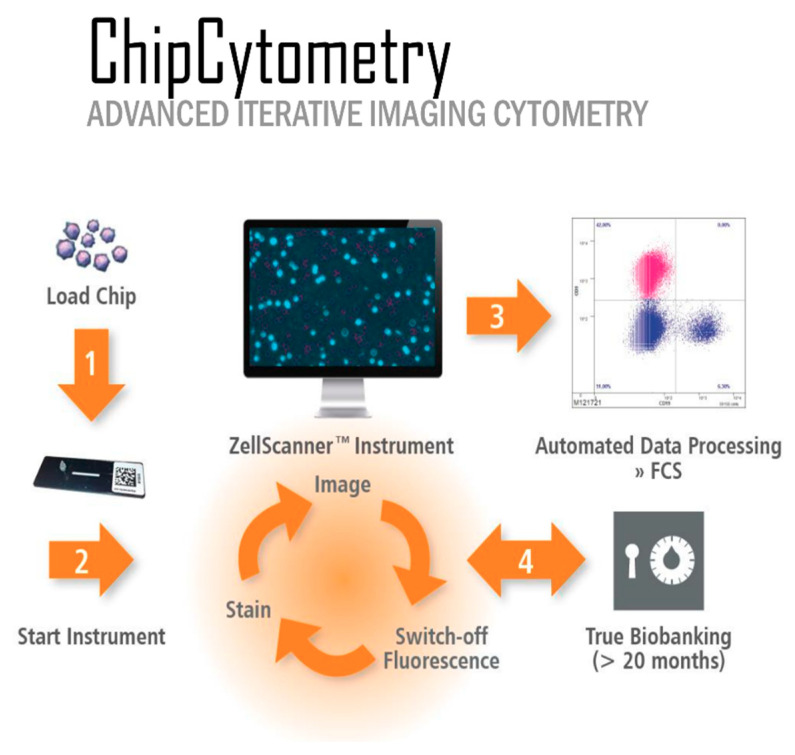
Workflow of Chipcytometry. Cells or tissue samples are loaded onto ZellSafe^TM^ chips (1) and fixed. After starting the process (2), repetitive cycles of staining up to five markers and photobleaching afterwards generates multiplex data that can be analyzed via the ZellExplorer Software and exported as Flow Cytometry Standard files (3). Furthermore, storage times of more than 20 months enable biobanking (4) and reanalysis if necessary or wanted (4).

**Figure A3:**
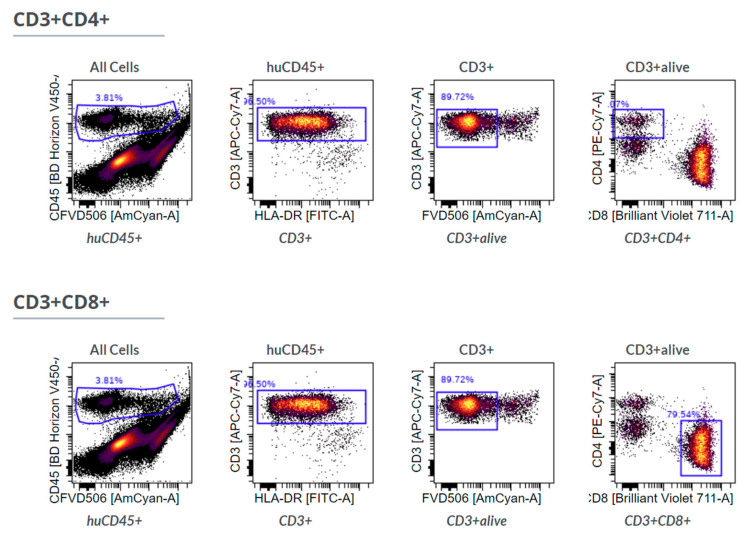
Gating strategy in flow cytometry to identify living CD4^+^ and CD8^+^ T cells in the digested tumor tissue. First, human immune cells are identified by human CD45 expression. Afterwards non-T cells (CD3^-^) and dead cells (FVD506 high) are excluded. Finally, cells are classified by either CD4 or CD8 expression and double negative or double positive cells are excluded.

Since the effects of Tasquinimod were only visible in the presence of human immune cells (Figure A1), we next analyzed the tumor microenvironment for immune cell markers in more detail using multiplex analysis.

**Figure 7:**
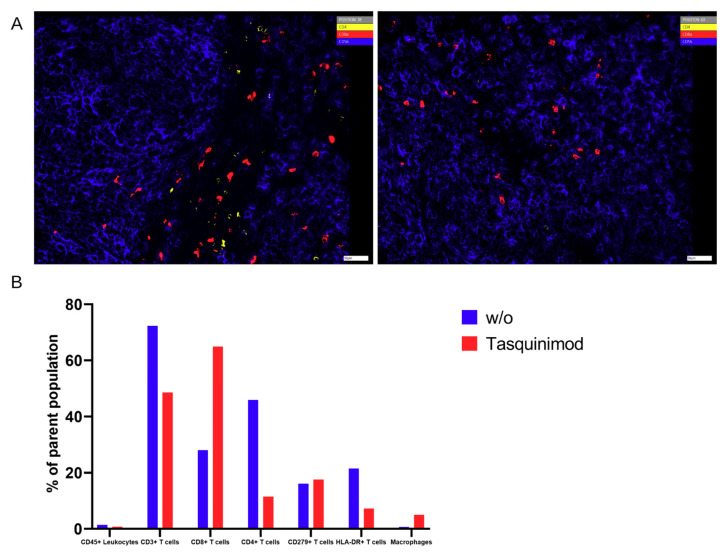
(**A**) Chipcytometry analysis showing exemplarily the identification of tumor-infiltrating CD8^+^ (red) and CD4^+^ (yellow) T cells within their spatial context with Tasquinimod (**right**) or without (**left**). (**B**) Percentage of cell populations in a representative section of the TME. One experiment out of two (*w*/*o*) or out of four (treatment) is shown.

Herein we detected significantly higher frequencies of CD8^+^ T cells in tumors of mice treated with Tasquinimod when compared to untreated tumor-bearing mice, which only showed CD8^+^ T cells in the marginal zones of the tumor (Figure 7A). Furthermore, more CD68^+^ macrophages were present in Tasquinimod treated tumors. In contrast, untreated mice displayed higher frequencies of CD4^+^, HLA-DR^+^, and CD279^+^ T cells in the tumor microenvironment (Figure 7B).

These results strengthen the idea, that the modulation of immune cells using systemic therapies is important for effective therapeutic responses, which can be analyzed in a comprehensive way on single cell level, as well as in the spatial context when using multiplex analysis, such as Chipcytometry. Our results are a first proof-of-concept that co-localization of cells and their organization in tissues constitute valuable information, especially in the context of different therapeutic approaches.

## 3. Discussion

The prognostic and therapeutic value of the tumor microenvironment (TME) in various cancer types is of major interest. Nevertheless, the complexity of the tumor microenvironment acts as a major obstacle in understanding immune regulation of anti-tumor responses, as well as responses to different immunotherapeutic strategies. As the number of clinical trials significantly increased in the last years and the development of markers predictive of therapy response continuously expands, there will be a growing need for (i) relevant preclinical models and (ii) adequate and comprehensive immunomonitoring of tissue specimens in preclinical, as well as in clinical studies.

Characterization of numerous immune cell subpopulations in a tissue sample is only possible with multiplex methods using 20 or more markers. Although fluidics-based systems like flow cytometry or mass cytometry do offer the required depth of markers, simple number games or intensity of markers do not offer the complete perception of cellular processes. Co-localization of cells and their organization in tissues constitute valuable information that is routinely lost during sample preparation. Multiplex immunohistochemistry or immunofluorescence methods leave spatial information of cells intact and deliver sufficient markers per cell at the same time.

Another aspect is the rarity of tissue samples derived either from animal experiments or from patients. Long time storage, labeling for multiple immunostainings, and several cycles of analysis in the very same cell or tissue section are gaining, thereby inestimably in value.

The herein used Chipcytometry technique offers those two characteristics mentioned and can be conveniently conducted with fluorescence-labeled antibodies that are used for flow cytometry or immunofluorescence. Of note, there are several other platforms, such as PerkinElmer or Roche, which use multi-epitope ligand cartography (MELC) to subject samples to multiple cycles of fluorescent staining, imaging, and photobleaching, very similar to the proposed technique.

In this work, we compared data obtained from three different methods in their ability to identify human immune cells in melanoma in vivo in a relevant preclinical model. Being aware that the three different methods described here are different in terms of sample preparation, all methods use the xenograft tumors as the tissue source. Chipcytometry uses frozen or paraffin-embedded tissue, flow cytometry uses viable or fixed cells, and IHC uses formalin-fixed paraffin-embedded tissue. The different preparation and fixation are mandatory to employ the three methods described in this study without changing the cell composition of the tissue. The main objective of the current study was to focus on the ability of each method to resolve human immune cells in the tumor microenvironment to identify the most suitable method for future studies using immunotherapeutic strategies and/or nanoparticle-based therapies.

In the present proof-of-concept study, we concentrated mainly on T cells and on macrophages being important effector cells in the tumor microenvironment. Nevertheless, this panel will be extended in future approaches.

IHC-P was limited to immune cells (huCD45^+^) and T cells (huCD3^+^) because staining using two different anti-human CD68 antibodies caused unspecific staining in the control xenografts without human immune cells (data not shown), showing the limitations of this approach. Furthermore, we stained for huCD4 and huCD8, but refrained from including those single positive cells in the further analysis as CD4^+^ or CD8^+^ T cells, since CD4 and CD8 expression is not exclusive for T cells [24,25].

The comparison showed that, in general, the results of those three methods are comparable without differences among those few markers that are accessible by all methods. At the end of the experiments in mice, T cells were the major immune cell type extracted. Those changes in contrast to the cell composition at the time of PBMC injection arose over time in the xenograft model.

Nevertheless, there was a significant discrepancy between flow cytometry and Chipcytometry regarding the ratio of CD8^+^ and CD4^+^ T cells. This ratio was far higher in flow cytometry than in Chipcytometry. One advantage of Chipcytometry is the preservation of spatial information. As in IHC-P, cell analysis is only performed in the tumor mass and not in the surrounding tissue. This is especially important as it is shown that T effector cells are rather localized in the marginal zones of the tumor and do not enter the tumor tissue itself. Detected immune cells in Chipcytometry, thus have been migrating actively into the tumor. In contrast, in flow cytometry, the whole tumor (including mouse skin, tissue and possibly the PBMC injection site) is digested and analyzed. In sum, Chipcytometry rather shows the T cell ratio inside the tumor, whereas flow cytometry shows the T cell ratio in the whole tissue sample.

Several different multiplexed tissue imaging techniques besides the fluorescent-based strategy (PerkinElmer, Roche, Zellkraftwerk), such as DNA barcoding-based (NanoString, Ultivue, Akoya) or metal-based (Fluidigm or IonPath) approaches, have been developed in the last years all of them providing a comprehensive view of the composition of the TME by labeling multiple markers on limited samples, thus also providing insight into tumor pathogenesis, as well as responsiveness to therapy [11]. Besides this, every technology has its limitations, such as being limited in the visualization of co-localized biomarkers, being time-consuming or costly, and thus, not applicable for every day.

The multiplex technique used in this study combines the advantages of flow cytometry and microscopy, offering the possibility to analyze more than 100 biomarkers consecutively on the same cell, also allowing the consecutive use of different staining protocols. In addition, it provides a platform that delivers the complete pipeline from staining to thigh dynamic range imaging to analysis. This allows the analysis of cells that can only be identified by marker combinations and the locating of rare cells in tissue samples. Although several new techniques of cell stabilization have been published in recent years, marker stability on and in cells remains very limited (maximum 12–72 h after sampling). Conventional flow cytometry requires fresh samples for biomarker development, and the cryopreservation of immune cells leads to significant changes in biomarker signatures. In Chipcytometry, the cells are fixed on coated glass surfaces, thereby yielding remarkable long-term stability of the surface and intracellular biomarkers (minimum 24 months after sampling), which is very useful for biobanking of precious or rare samples. In contrast to conventional flow cytometry, where samples are lost during analysis, samples are stored on small microfluidic chips, where they can be reused and assayed several times again with new and additional markers at any time new or additional questions are to be addressed.

Limitations of our study clearly includes the small sample size, as well as it’s exploratory nature. To analyze melanoma tumor micromilieu, the tissue had to be prepared in different ways. Due to this and the limited material, tumors were used from the same experimental groups. Nevertheless, we could only compare the same samples for flow cytometry and immunohistochemistry. We also did not assess further therapy outcomes with Tasquinimod on cellular levels in more depth. As tumor volume was significantly reduced in Tasquinimod treated animals, we were not able to extract enough tissue to perform a comprehensive comparative analysis for cellular composition and spatial distribution, which is a question we would like to expand on in future studies with adequate sample sizes.

To sum up, depending on the goal of the clinical or research questions, all techniques have their pros and cons. Thus, for each study or experiment, it is necessary to carefully consider which method is suited best. The present study wanted to point out the advantages of every single method and its possible application in preclinical and clinical studies.

Clearly, in routine pathology (where few single cell markers can be enough to identify certain malignancies) classical IHC-P is sufficient. This method is commonly used as an important diagnostic, highly practical, available, and cost-effective diagnostic and prognostic device by staining one or two markers, being able to deliver spatial information. Nevertheless, IHC does allow for multiplex staining. Without an extensive marker panel, it misses the opportunity to gain important information from patient samples and their complex immune microenvironment also in the long term run in both research and clinical settings.

Flow cytometry has its power in cell throughput, the possibility to sort and to detect rare cells with a high marker resolution when spatial information is not mandatory, e.g., blood samples. In tissues, it does not consider the tissue architecture. Thus, an in-depth analysis of different areas of interest in the tumor microenvironment is not possible.

Each time spatial information must not be lost, and high amounts of cellular markers are needed simultaneously, like in tumor biopsies, multiplex methods that can be employed on tissue samples are superior to IHC-P regarding multiplex and to flow cytometry in regard to spatial information.

Chipcytometry delivers as a single method more information in the very same tissue section than both of the other methods, IHC-P and flow cytometry, combined (Figure 3), and thus, is a powerful discovery tool with the ability to investigate the phenotype of rare immune cells in the TME, thus allowing comprehensive studies of cell composition, cell–cell interaction and cellular function with a potential diagnostic benefit. It offers quantitative data for tissue analysis comparable to flow cytometry, while also providing an “IHC-like”, histological view of the tissue structure. It is, therefore, suitable for in-depth immuno-oncology studies, including different therapeutic approaches. Another advantage compared to flow cytometry is that cells once fixated can be analyzed for up to another 20 months with additional markers at any time. Chipcytometry is mainly limited by its availability and cost limitations since staining, and data acquisition requires automation.

Importantly, in the current study, we did not want to address different treatment modalities. We wanted to compare different analysis methods to study in-depth the tumor microenvironment in an adequate preclinical model, which subsequently, in further studies, allows translation into the clinical settings.

To our knowledge, this is the first approach to combine a relevant preclinical humanized mouse model with different methods to analyze TME. In our hands, the introduced human melanoma cells establish a tumor environment relatively close to the known environment of human melanomas. Cytokines and chemokines produced within the TME attract human immune effector cells being the targets for immunological treatment strategies. First hints are given by the results of the Tasquinimod treatment group, herein only mice substituted with human PBMC showed tumor regression. In further studies, this approach will help to further validate different treatment modalities, especially in the context of immunotherapeutic strategies, where cellular compositions, and more importantly, their distribution (cold and hot tumors) play an important role. Of note, tumors can have the same overall cellular composition (e.g., shown in flow cytometry), but in cold tumors immune cells do not invade the tumor itself in contrast to hot tumors. Therefore, the spatial information, in our opinion, is of great relevance to validate therapeutic responses, and thus, was the focus of our study.

Further development of the multiplexed techniques resulting in high-throughput and standardized quantitative analysis for high reproducibility, as well as better cost-effectiveness, will result in methods with great potential in translational research defining prognostic or predictive cellular biomarkers in different tumor entities with or without treatment.

## 4. Materials and Methods

### 4.1. Mice

Animal experiments were approved by local authorities (G 15-1-070, Landesuntersuchungsamt Rhineland-Palatinate, Germany). NOD.Cg-*Mcph1^Tg(HLA-A2.1)1Enge^Prkdc^scid^*/DvsJ mice (#006609) acquired from The Jackson Laboratory (Bar Harbor, USA) were injected subcutaneously (s.c.) with 2 × 10^6^ Ma-Mel-19 melanoma cells at an age older than 8 weeks. Tumor volume was measured using a caliper and the formula V = (length/2) × (width^2^). After randomization 20 × 10^6^ human PBMC were injected s.c. and intraperitoneally (i.p.) each. Tasquinimod (#S7617, Selleckchem, Munich, Germany) was applied at doses of 5 mg/kg or 1 mg/kg weekly s.c. at the tumor site in a 1:4 mixture of DMSO (#D2650, Merck, Darmstadt, Germany) and NaCl solution (0.9%) (#3200910, B. Braun, Melsungen, Germany). The control group received the mixture without the compound. After 3 weeks of treatment, the animals were sacrificed, and ex vivo analysis of the tumors was performed. All animals were housed under specific pathogen-free conditions in the central animal facility of the Johannes Gutenberg-University in Mainz, and experiments were performed in accordance with relevant laws and institutional guidelines.

### 4.2. Cell Lines and Cell Culture

The Ma-Mel-19 cell line originates from a (sub)cutaneous biopsy of stage IV superficial spreading melanoma from a 62-year-old female patient, harbors a B-Raf V600E mutation, and is N-Ras wild-type [26]. The cell line has been tested and authenticated at Leibniz Institute (DSMZ) in Braunschweig, Germany, using DNA profiling lastly in May 2020. Generated STR profiles were matching the STR reference profile of respective parental cell lines from cell banks ATCC, HPACC, JCRB, RIKEN, KCLB, EMBL, and DSMZ. The cell line was cultured with RPMI-1640 (#31870, Thermo Fisher Scientific, Waltham, MA, USA) supplemented with 10% FBS (#10500064, Thermo Fisher Scientific, Waltham, MA, USA), 1% GlutaMAX™ (#35050038, Thermo Fisher Scientific, Waltham, MA, USA) and 0.1% primocin (#ant-pm-2, InvivoGen, San Diego, CA, USA). Cells were detached via Trypsin-EDTA (#T3924, Merck, Darmstadt, Germany) for 5 min every 3 to 4 days. The Ma-Mel-19 cell line was authenticated at Eurofins Genomics (Ebersberg, Germany) in May 2020. The resulting STR profiles were matched with the online databases of the German collection of microorganisms and cell cultures (http://www.dsmz.de/de/service/services-human-and-animal-cell) and Cellosaurus database (https://web.expasy.org/cellosaurus/) references.

### 4.3. PBMC Isolation

Buffy coats were obtained from healthy volunteers, with approval by the local ethical committee (Landesärztekammer Rhineland Palatine No. 837.019.10 (7028), approved on 4 March 2010). PBMC were isolated using density gradient centrifugation with Biocoll Separating Solution (#L6115, Merck, Darmstadt, Germany).

### 4.4. Flow Cytometry

Tissue samples were digested by incubation with Accumax (#00-4666-56, Thermo Fisher Scientific, Waltham, MA, USA) for 1 h at room temperature followed by manual disruption and passage through a 40 μm Cell strainer (#732-2757, VWR International, Radnor, PA, USA). Single cell suspensions were treated either with Foxp3/Transcription Factor Staining Buffer Kit (#00-5523-00, Thermo Fisher Scientific, Waltham, MA, USA) (T cell panel) or fixed with 1% PFA (#0335.1, Carl Roth, Karlsruhe, Germany) in DPBS (#14190-094, Thermo Fisher Scientific, Waltham, MA, USA) and stained in 20 µL/sample FACS buffer containing 0.5% HSA (#10530a/96, CSL Behring, Marburg, Germany), 1 mM EDTA (#A3553, AppliChem, Darmstadt, Germany), 10 μg/mL human IgG (#EU/1/08/446/001, CSL Behring GmbH, Marburg, Germany) in DPBS (#14190-094, Thermo Fisher Scientific, Waltham, MA, USA).

The following antibodies were used to stain cells in flow cytometry experiments: Anti-human CD3-APC-Fire750 (#344840, BioLegend, San Diego, CA, USA), anti-human CD4-PE-Vio770 (#130-100-454, Miltenyi Biotech, Bergisch Gladbach, Germany), anti-human CD45-VioBlue (#130-092-880, Miltenyi Biotech, Bergisch Gladbach, Germany), anti-human CD68-Brilliant Violet 711 (#565594, BD Biosciences, Franklin Lakes, NJ, USA), anti-human CD8-BV711 (#344734, BioLegend, San Diego, CA, USA) and fixable viability dye eFluor™ 506 (#65-0866-14, Thermo Fisher Scientific, Waltham, MA, USA). All antibodies were used in 20× dilutions, the viability dye in 200× dilution.

Flow cytometry was performed on an LSRII flow cytometer (BD Biosciences, Franklin Lakes, NJ, USA), and data were analyzed using Cytobank [27].

### 4.5. Immunohistochemistry

Tumor specimens were immunohistochemically analyzed for infiltration with CD45^+^ and CD3^+^ immune cells. Tumor sections (4 µm) were prepared from formalin-fixed, paraffin-embedded tissue. After de-paraffinization and rehydration, slides were boiled. For CD45 stains, slides were boiled in Target Retrieval Solution, Low pH (#K800521-2, Agilent, Santa Clara, CA, USA). For CD3, CD4, and CD8 stains, slides were boiled in Target Retrieval Solution, High pH (#K800421-2, Agilent, Santa Clara, CA, USA). Endogenous peroxidase activity was blocked by Peroxidase-Blocking Solution (#S2023, Agilent, Santa Clara, CA, USA) for 5 min. Sections were blocked with Normal Horse Serum Blocking Solution (#S-2000-2, Vector Laboratories, Burlingame, CA, USA). Anti-human CD45 (clone M0701, Agilent, Santa Clara, CA, USA), anti-human CD3 (#NCL-LCD3-565, Leica Biosystems Nussloch GmbH, Nussloch, Germany), anti-human CD8 (clone M7103, Agilent, Santa Clara, CA, USA) and anti-human CD4 (#104R-26, Merck, Darmstadt, Germany) were applied as primary mAb. For CD3 and CD45, slides were incubated with a secondary biotinylated Horse Anti-Mouse IgG Antibody (#BA-2000-1.5, Vector Laboratories, Burlingame, USA). The antigen detection by a color reaction with 3,3′-diamino-benzidine (#K346711-2, DAB+, Agilent, Santa Clara, CA, USA) catalyzed by VECTASTAIN^®^ Elite ABC-HRP Reagent, Peroxidase, R.T.U. (#PK-2000-2, Vector Laboratories, Burlingame, CA, USA). For CD4 and CD8, samples were stained with EnVision Detection SystemsPeroxidase/DAB, Rabbit/Mouse (#K5007, Agilent, Santa Clara, CA, USA). Both methods were counter stained with Mayer’s hemalum solution (#109249, Merck, Darmstadt, Germany).

Tissue samples were imaged by the tissue bank of the University Medical Center Mainz using a Hamamatsu NanoZoomer 2.0HT. IHC data were analyzed using QuPath [28].

### 4.6. Multiplex Staining

Tissue sections with 7 µm thickness were prepared at a standard cryostat and mounted on glass coverslips. The sections were fixed by immersion in ice-cold 100% acetone (#9372.1, Carl Roth, Karlsruhe, Germany) for 5 min, followed by serial immersion in 90% ethanol (#5054.1, Carl Roth, Karlsruhe, Germany), 70% ethanol, and PBS for 3 min at 4 °C, respectively. The glass coverslips with the fixed tissue samples were loaded onto ZellSafe^TM^ Tissue Chips (#28050606/02-010, Zellkraftwerk, Leipzig, Germany), chips were filled up with storage buffer (Zellkraftwerk, Leipzig, Germany) and stored at 4 °C until and in between staining cycles.

The analysis was performed on a ZellScanner One^®^ instrument (Zellkraftwerk, Leipzig, Germany). Each of the 3 tissue samples was stained and imaged with an iterative multiplex staining assay summarized in Table 2. Tissue chips were rinsed with 5 mL of wash buffer before starting an imaging cycle consisting of photobleaching for 40 s per scanned position, followed by imaging of tissue autofluorescence and subsequently antibody staining and imaging of fluorescence signal. Of note, there is no antibody or fluorophore detachment step. Background fluorescence is recorded before each acquisition step. Staining was performed by diluting the antibodies in the storage buffer and pipetting the working solution into the chip flow chamber. After incubation for 15 min at room temperature, the chip was rinsed thoroughly with wash buffer before imaging. This process was repeated until all biomarkers were stained and imaged. In one of the cycles, the cell nuclei were stained by incubating with 0.05 µg/µL Hoechst 33342 (#H3570, Thermo Fisher Scientific, Waltham, USA) in storage buffer for 5 min at room temperature.

In total, analysis on tumor tissue using Chipcytometry was performed six times (n = 2 tumor without treatment, n = 4 tumor with treatment).

The resulting images were analyzed with ZellExplorer data analysis software (Zellkraftwerk, Leipzig, Germany). Net-fluorescence images were generated by subtracting the autofluorescence from the staining fluorescence image for each cycle and position. Cell segmentation was performed based on the nuclear DNA stain, and for each segmented cell, the fluorescence values for each marker stained in the multiplex assay were calculated, resulting in a single cell resolution quantitative data set. This data set was analyzed by employing a gating strategy to identify cell populations of interest-based on biomarker expression [12].

### 4.7. Statistics

Statistical calculations were performed by GraphPad Prism V6 (GraphPad Software, San Diego, CA, USA). Results were normalized to the untreated samples as indicated. Box and whiskers plots display median with the 25th and 75th percentiles and min to max whiskers. Statistical significance was determined using Tukey’s multiple comparison test and Šídák’s multiple comparison test as indicated in the figure legends with * *p* < 0.05, ** *p* < 0.01, *** *p* < 0.001, **** *p* < 0.0001.

## 5. Conclusions

The present study demonstrates the advantages and disadvantages of each method, and its possible application in preclinical and clinical studies. The great and complementary potential of multiplex marker analysis of tumor tissue should be considered for both scientific questions and diagnostic purposes. As the cellular composition in a tumor, and its microenvironment, is of significant importance for disease progression, prognosis and therapeutic approaches, these techniques help develop, monitor, and validate new therapeutic strategies.

## Figures and Tables

**Table 1:** Immune cell subpopulations in untreated tumor-bearing animals detected by Chipcytometry (n = 3).

Marker Definition	Parent Population	Cell Type	% of Parent Population	SD
CD45+	All	immune cells	6.7	5.7
CD3+	CD45+	T cells	74.8	7.1
CD4+	CD45+CD3+	T helper cells	31.1	11.7
CD8+	CD45+CD3+	cytotoxic T cells	30.9	6.7
CD45RO+CD45RA-	CD45+CD3+CD4+	activated/memory t cells	85.2	20.1
CD45RO+CD45RA-	CD45+CD3+CD8+	activated/memory t cells	84.0	24.2
CD4+FOXP3+CD25+	CD45+CD3+CD4+	regulatory T cells	4.8	3.3
CD279+	CD45+CD3+CD4+	PD1+ T cells	24.8	10.3
CD366+	CD45+CD3+CD4+	TIM-3+ T cells	59.2	29.8
CD279+	CD45+CD3+CD8+	PD1+ T cells	16.5	9.7
CD366+	CD45+CD3+CD8+	TIM-3+ T cells	54.7	5.1
CD68+	CD45+	macrophages	1.3	0.9

**Table 2:** Multiplex staining Chipcytometry.

No.	Cycle	Marker	Clone	Catalog Number	Fluorophore	Vendor
1	1	CD25	M-A251	555432	PE	BD Biosciences
2	2	FOXP3	236A/E7	12-4777-42	PE	Thermo Fisher Scientific
3	3	CD3	UCHT1	563546	BUV395	BD Biosciences
4	3	CD4	RPA-T4	300530	PerCP-Cy5.5	Biolegend
5	3	CD8	RPA-T8	301008	PE	Biolegend
6	4	CD14	HCD14	325622	PerCP-Cy5.5	Biolegend
7	4	CD56	AF12-7H3	130-113-307	PE	Miltenyi Biotech
8	4	CD68	KP1	sc-20060	AF488	Santa Cruz
9	5	CD45	HI30	304028	PerCP-Cy5.5	Biolegend
10	5	CD45RO	REA611	130-113-559	PE	Miltenyi Biotech
11	5	CD45RA	HI100	740298	BUV395	BD Biosciences
12	6	DNA	---	H3570	BUV395+BV421	Thermo Fisher Scientific
13	7	HLA-DR	L243	307606	PE	Biolegend
14	8	CD279	EH12.1	560795	PE	BD Biosciences
15	9	CD86	2331 (FUN-1)	555658	PE	BD Biosciences
16	10	CD105	43A3	323206	PE	Biolegend
17	11	CD366	7D3	563422	PE	BD Biosciences

## Data Availability

The data that support the findings of this study are available from the corresponding author upon reasonable request.
